# A signature based on 11 autophagy genes for prognosis prediction of colorectal cancer

**DOI:** 10.1371/journal.pone.0258741

**Published:** 2021-10-26

**Authors:** Shuo Chen, Yan Wang, Boxue Wang, Lin Zhang, Yinan Su, Mingyue Xu, Mingqing Zhang

**Affiliations:** 1 Department of colorectal Surgery, Tianjin Union Medical Center, Tianjin, P.R. China; 2 Tianjin Institute of Coloproctology, Tianjin, P.R. China; 3 Department of Traditional Chinese Medicine, Shanghai Pudong New Area People’s Hospital, Shanghai, P.R. China; Howard University, UNITED STATES

## Abstract

**Aim:**

To develop an autophagy-gene-based signature that could help to anticipate the therapeutic effects of Colorectal Cancer (CRC).

**Methods:**

We downloaded the gene expression profiles of CRC samples from the Cancer Genome Atlas (TCGA) and the Gene Expression Omnibus (GEO) datasets. Genes with significant prognostic value in CRC were screened through univariate Cox regression analysis, while the LASSO Cox regression method was applied to screen optimal genes to construct the autophagy‐related prognostic signature.

**Results:**

11 autophagy genes were identified and selected for the establishment of prognosis prediction model for CRC patients. The CRC patients were classified into the low- and high-risk groups according to the optimal cutoff value. The time-dependent ROC curves indicated the good performance of this model in prognosis prediction, with AUC values of 0.66, 0.66, and 0.67 at 1, 3 and 5 years for TCGA samples, as well as AUC values of 0.63, 0.65 and 0.64 for GEO samples, respectively. The multivariate Cox regression analysis results confirmed risk score as the independent marker for prognosis prediction in CRC. Besides, the constructed nomogram also had high predictive value. The results analysis on the tumor infiltrating immune cells (TIICs) relative ratios and mRNA levels of key immune checkpoint receptors indicated the signature was closely related to immune microenvironment of CRC in the context of TIICs and immune checkpoint receptors’ mRNA level. The proportion of MSI-L + MSI-H in the high-risk group was higher than that in the low-risk group. Moreover, the tumor purity was evaluated by estimate function package suggested that lower tumor purity in CRC might lead to a poorer prognosis.

**Conclusion:**

The autophagy-related features obtained in this study were able to divide the CRC patients into low- and high-risk groups, which should be contribute to the decision-making of CRC treatment.

## Introduction

According to statistical report in 2019, colorectal cancer (CRC) is one of the most common cancers in Chinese population [1]. The CRC incidences in several areas of China are on the rises, and approximately 50% of patients are diagnosed in the middle and late stages [2]. Although surgical therapy remains the cornerstone in the treatment of CRC, recurrence and metastasis still occur after operation. Other approaches including radiotherapy, chemotherapy, targeted therapy, and immunotherapy, have shown clinical benefit in enhancing the control rate in CRC patients with local tumor, as well as extending the survival of CRC patients with metastasis [3]. The treatment should be selected after comprehensively considering the disease onset characteristics, disease development trends and the current domestic and international guidelines [4]. However, there is still lacking an effective method for estimating the prognosis of CRC patients after treatments [5].

Recent studies have revealed that, over 70% of the CRC cases are sporadic with somatic mutations, whereas the others are associated with a family history or colorectal hereditary diseases [6]. The genetic and epigenetic alterations induce the colon cancer formation and promote the cancer development. Autophagy often occurs when the transformation of cells from normal to malignant appears in CRC [7]. It has been known that the molecular network of autophagy can clinically, biologically, and mechanistically control CRC chemoresistance [8]. Moreover, the autophagy gene ATG16L1 +898A>G polymorphism can affect the CRC risk, and for male patients, the GG-genotype carriers have a high risk of CRC [9]. Consequently, investigation on the association between autophagy genes and CRC may be conducive to elucidate the underlying function of autophagy genes in CRC.

In this study, we proposed to explore the underlying relationship between the autophagy-associated gene expression and prognosis of CRC, aiming to establish an efficient prognostic signature based on autophagy genes. The risk score calculated by this gene expression-based signature was able to predict the CRC prognosis independently, and the impact of these genes on tumor heterogeneity and immune microenvironment were both preliminary explored.

## Materials and methods

### Study population

We downloaded the mRNA expression profiles and clinical information of 622 colon and rectal cancer patients from the Cancer Genome Atlas (TCGA, https://tcga-data.nci.nih.gov/tcga/), in which 590 samples had complete survival information (Table 1). The dataset numbered GSE17536 which contained the expression profiles of 177 CRC patients (Affymetrix Human Genome U133 Plus 2.0 Array) were obtained from the Gene Expression Omnibus (GEO, https://www.ncbi.nlm.nih.gov/geo/), and 172 out of those 177 samples had complete survival information. Besides, we obtained the 210 autophagy genes from the website www.autophagy.lu/project.html, which were provided in S1 Table.

**Table 1:** Clinicopathological features of colon and rectal cancer patients in TCGA dataset.

Characteristics		Patients(N = 590)	
		NO.	%
**Sex**	**Female**	269	45.59%
	**Male**	321	54.41%
**Age**	**≤67(Median)**	296	50.17%
	**>67(Median)**	294	49.83%
**Race**	**white**	288	48.81%
	**Black or african american**	62	10.51%
	**asian**	12	2.03%
	**American indian or alaska**	1	0.17%
	**Unknown**	227	38.47%
**Pathologic stage**	**i**	103	17.46%
	**ii**	212	35.93%
	**iii**	170	28.81%
	**iv**	85	14.41%
	**Unknown**	20	3.39%
**Survival Time**	**Long(>5 years)**	48	8.14%
	**Short(<5 years)**	542	91.86%
**OS status**	**Dead**	122	20.68%
	**Alive**	468	79.32%

### LASSO Cox regression analysis

Autophagy genes related to CRC prognosis were preliminary selected by univariate Cox-regression analysis based on the expression values of 210 autophagy genes with P value less than 0.05 as the threshold, which were further optimized by LASSO Cox-regression analysis using glmnet function package in R [10]. We computed the risk score with the following formula:

$$Risk\;score=\sum_{i=1}^{n}Coef_{i}*x_{i}$$

*Coef_i_* was defined as the coefficient of factors computed by LASSO-Cox model, while *x_i_* was defined as the mRNA expression levels of factors. We measured the optimal cutoff value of risk score using survival and survminer packages, and two-sided log-rank test in R software. Then we stratified the CRC patients into high- and low-risk groups based on the cutoff value.

### Survival analysis

Kaplan-Meier method was applied to evaluate the survival probability of CRC patients using survival and survminer packages, and the difference in survival between two groups were assessed by log-rank test. The time-dependent receiver operating characteristic (ROC) curve was plotted using survivalROC package [11]. To evaluate whether risk score was an independent predictive signature for CRC prognosis, multi-factor Cox regression analysis was carried out.

### Calculation of tumor infiltrating immune cells and tumor purity

The relative proportions of 22 tumor infiltrating immune cells (TIICs) were estimated by using CIBERSORT [12], which is a gene expression-based method that characterizing the TIICs composition with deconvolution algorithm based on the pre-set 547 barcode genes. The estimate package in R software was adopted for computation of tumor purity [13].

### Nomogram analysis

Nomogram is widely applied to estimate the overall survival (OS) at 1, 3 and 5 years for CRC patients. Here, according to the independent prognostic factors selected by multivariate Cox regression analysis, we conducted the Nomogram analysis with the rms package in R software. The deviation between estimated and actual OS was estimated by the calibration curve.

### Functional enrichment analysis

The clusterProfiler package was used to perform the functional enrichment analysis. Benjaminiand Hochberg (BH) adjusted P value < 0.05 was considered as the threshold to select the significantly enriched Gene Ontology (GO) terms (including Biological Process, Molecular Function and Cellular Component) and Kyoto Encyclopedia of Genes and Genomes (KEGG) pathways.

### Statistical analysis

The OS probability of samples was calculated by Kaplan-Meier method, which was compared between different groups through log-rank test. Statistical comparison of TIICs between different groups was conducted using Wilcoxon rank sum test, with p < 0.05 as the threshold. All statistical analysis was completed using R software (version 3.5.2).

## Results

### Construction and verification of autophagy gene-based predictive model for CRC prognosis

**Fig 1:**
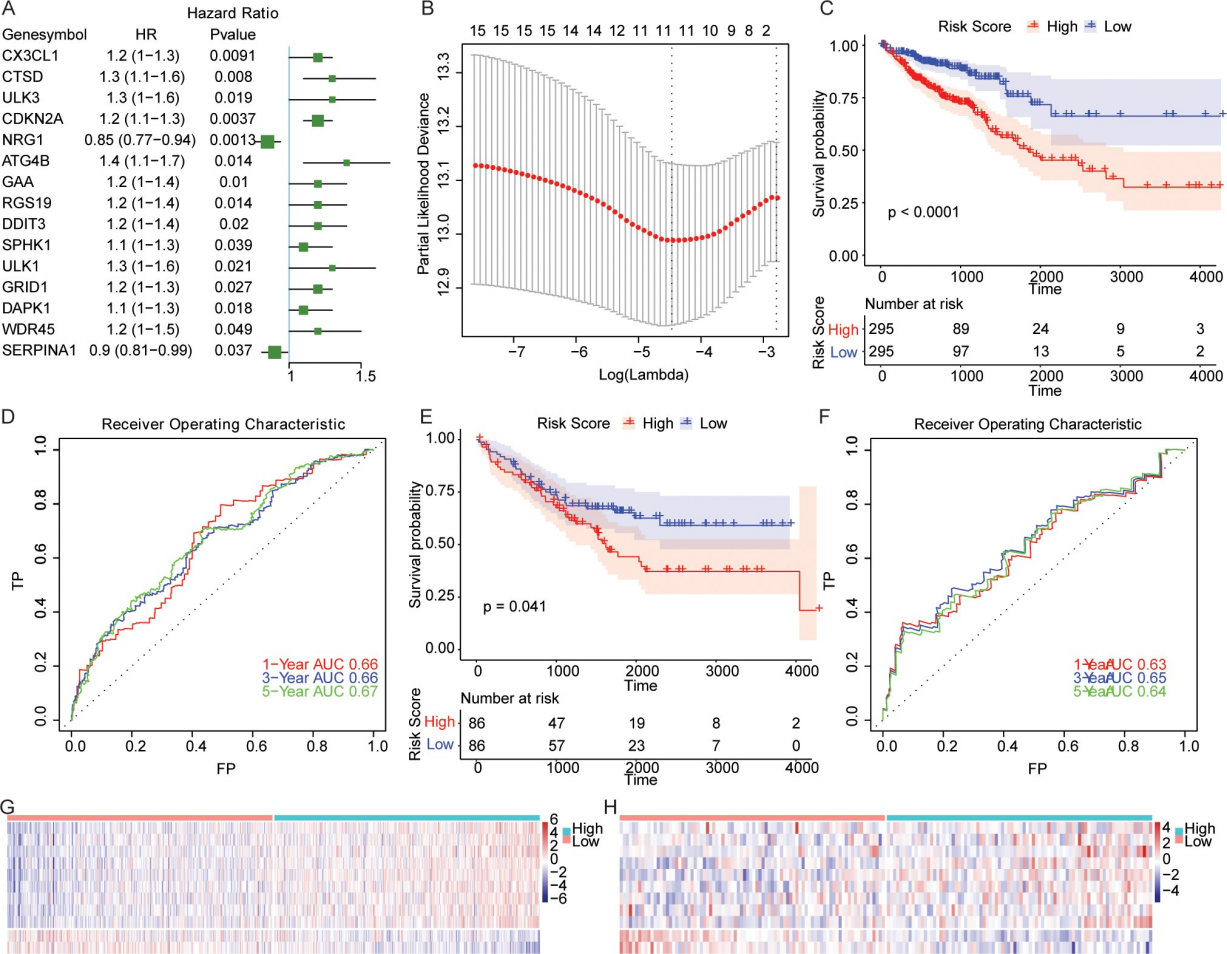
Construction of predictive model for CRC prognosis. (A) Univariate cox regression analysis of the 15 genes that were related to the overall survival (OS) of patients with CRC. HR: hazard ratio. (B) Determination of the lambda value in LASSO regression model. The lambda value which corresponded to the minimum value of Partial Likelihood Deviance was considered as the optimal one. (C) Survival curves of CRC patients in TCGA dataset. P value that calculated using log-rank test was provided. (D) Time-dependent ROC curve for the patients in TCGA dataset. TP: True Positive; FP: False Positive. (E) Survival curve of CRC patients in GEO dataset. (F) Time-dependent ROC curve for the patients in GEO dataset. TP: True Positive; FP: False Positive. (G-H) Heatmaps showing the differential expressions of *CX3CL1*, *ULK3*, *CDKN2A*, *NRG1*, *ATG4B*, *GAA*, *RGS19*, *DDIT3*, *GRID1*, *DAPK1* and *SERPINA1* of the samples in two groups stratified by risk score from TCGA and GEO respectively. Horizontal and vertical axis represents samples and genes, respectively. Red and blue dot represents higher and lower expressions respectively.

The functional enrichment analysis showed that the 210 autophagy related genes were significantly enriched in 1868 GO terms including macroautophagy and 139 KEGG pathways including autophagy-animal (S2 Table). The top 20 enriched GO terms and KEGG pathways were illustrated in S1 Fig. The expression values of the 210 autophagy genes were adopted as continuous variables in the univariate cox regression analysis of cases from TCGA dataset, and the Hazard Ratio (HR) values were computed. Finally, a total of 15 genes including *CX3CL1*, *CTSD*, *ULK3*, *CDKN2A*, *NRG1*, *ATG4B*, *GAA*, *RGS19*, *DDIT3*, *SPHK1*, *ULK1*, *GRID1*, *DAPK1*, *WDR45* and *SERPINA1* were screened out using the P value less than 0.05 as significant threshold (Fig 1A). The HRs of *NRG1* and *SERPINA1* were both less than 1, which was considered as protective genes and was beneficial to the prognosis, while the HR of the other 13 genes was greater than 1, indicating their high expressions could be associated with poor prognosis.

These 15 genes were further optimized by using LASSO Cox regression analysis. After evaluating the lambda values corresponding to different gene numbers (Fig 1B), *CX3CL1*, *ULK3*, *CDKN2A*, *NRG1*, *ATG4B*, *GAA*, *RGS19*, *DDIT3*, *GRID1*, *DAPK1* and *SERPINA1* were used to construct the model to predict survival in patients. The regression model was built as an equation: Risk Score = (Express Value of CX3CL1 * 0.01600218) + (Express Value of ULK3 * 0.03438969) + (Express Value of CDKN2A * 0.06438785) + (Express Value of NRG1 * -0.13918950) + (Express Value of ATG4B * 0.05534852) + (Express Value of GAA * 0.05028759) + (Express Value of RGS19 * 0.01419570) + (Express Value of DDIT3 * 0.00395248) + (Express Value of GRID1 * 0.06085908) + (Express Value of DAPK1 * 0.06678703) + (Express Value of SERPINA1 * -0.08109360).

Based on the cutoff value, we assigned the samples from TCGA and GEO datasets into low- and high-risk groups. As shown in Fig 1C and 1E, the patients with high risk score exhibited worse prognosis compared with patients with low risk score. The results of time-dependent ROC analysis for TCGA samples (Fig 1D) showed that, the AUC values for OS at 1, 3 and 5 years were 0.66, 0.66, and 0.67, while 0.63, 0.65 and 0.64 for GEO cohort (Fig 1F). Moreover, these 11 genes in the established model showed significantly different expressions between the low- and high-risk groups (Fig 1G and 1H). Overall, this risk model could efficiently forecast the survival outcome of CRC patients in both TCGA and GEO datasets.

### Potential of autophagy‐related signature in the survival prediction

**Fig 2:**
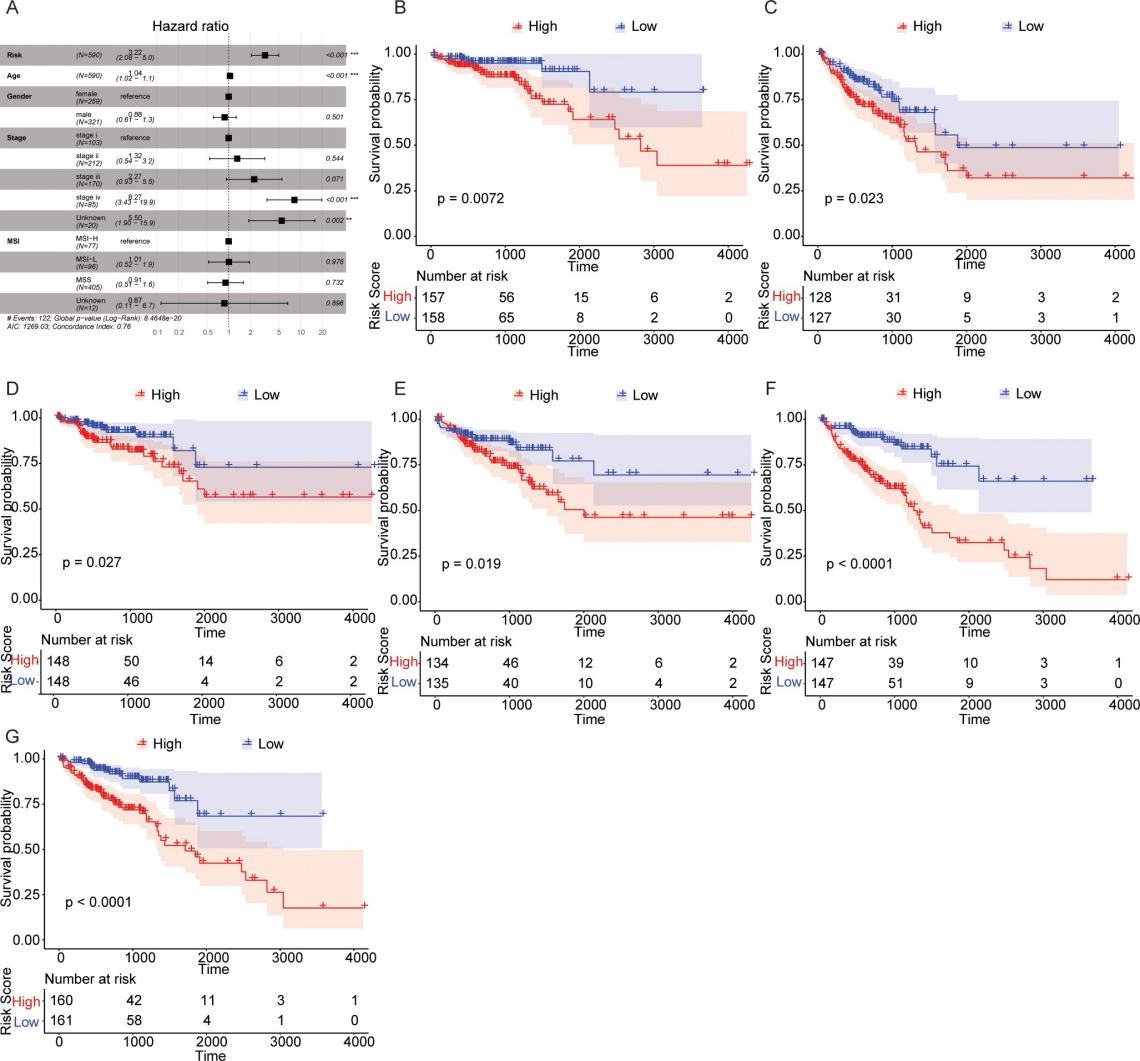
Risk score is a signature which could predict CRC prognosis independently. (A) Multivariate Cox regression analysis indicated samples with HR greater than 1 was related to a higher death risk, while samples with HR less than 1 exhibited lower risk of death. (B) Survival curves of CRC patients of Stage I+StageII stratified by risk score. (C) Survival curves of CRC patients of StageIII+StageIV stratified by risk score. (D) Survival curves of CRC patients less than 67 years old stratified by risk score. (E) Survival curves of CRC patients more than 67 years old stratified by risk score. (F) Survival curves of male CRC patients stratified by risk score. (G) Survival curves of female CRC patients stratified by risk score.

To explore whether risk score was an independent prognosis indicator in CRC, we performed multivariate Cox regression analysis by simultaneously taking multiple clinicopathological features such as age, TNM stage, gender, MSI status and risk score into account. As shown in Fig 2A, risk score was obviously associated with the OS of CRC patients (HR = 3.26, 95%CI: 2.11–5.04, P < 0.001). Moreover, we further evaluated the predictive significance of risk score in CRC prognosis among samples with various clinicopathological features. The patients were divided into different groups according to TNM stage (Fig 2B and 2C), age (Fig 2D and 2E), and gender (Fig 2F and 2G), and the survival analysis indicated significantly inferior OS of samples with higher risk score. The above results suggested risk score was able to predict the prognosis of CRC patients independently.

**Fig 3:**
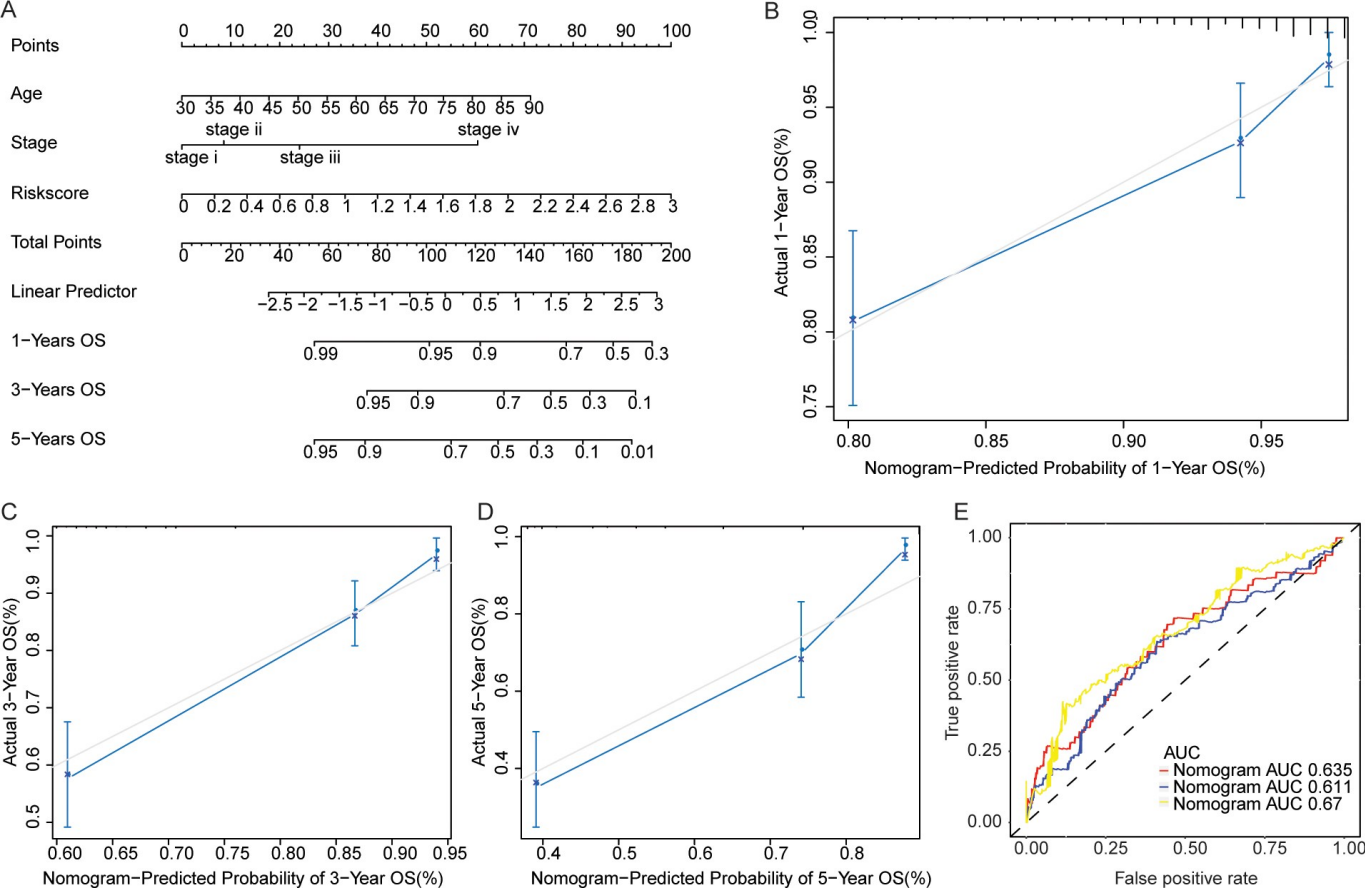
Nomogram for CRC survival outcome estimation. (A) Nomogram that combined TNM stage and risk score. A single point is assigned to the stage or risk score that is perpendicular to the points line. Total point is assigned to every CRC sample by combining the sample’s risk score and TNM stage corresponded point. The OS at 1, 3 and 5 years was estimated according to the corresponding total point. (B-D) Calibration curve for the nomogram which estimated the OS at 1, 3, and 5 years respectively. (E) Time-dependent ROC curve of the samples. The horizontal axis was the false positive rate, and the vertical axis was the true positive rate. The predictive accuracy was evaluated by AUC.

The nomogram model was established based on independent prognostic factors including age, TNM stage and risk score (Fig 3A). Calibration curve indicated that the deviation is very small between the actual and nomogram estimated OS of CRC patients at 1 (Fig 3B), 3 (Fig 3C), and 5 years (Fig 3D). Moreover, the time-dependent ROC curves revealed that the AUC values for 1, 3, and 5 years were all greater than 0.6 (Fig 3E), indicating that the constructed nomogram model was relatively reliable.

Potential of autophagy gene-based marker in the prediction of immunity therapy and precision treatment.

**Fig 4:**
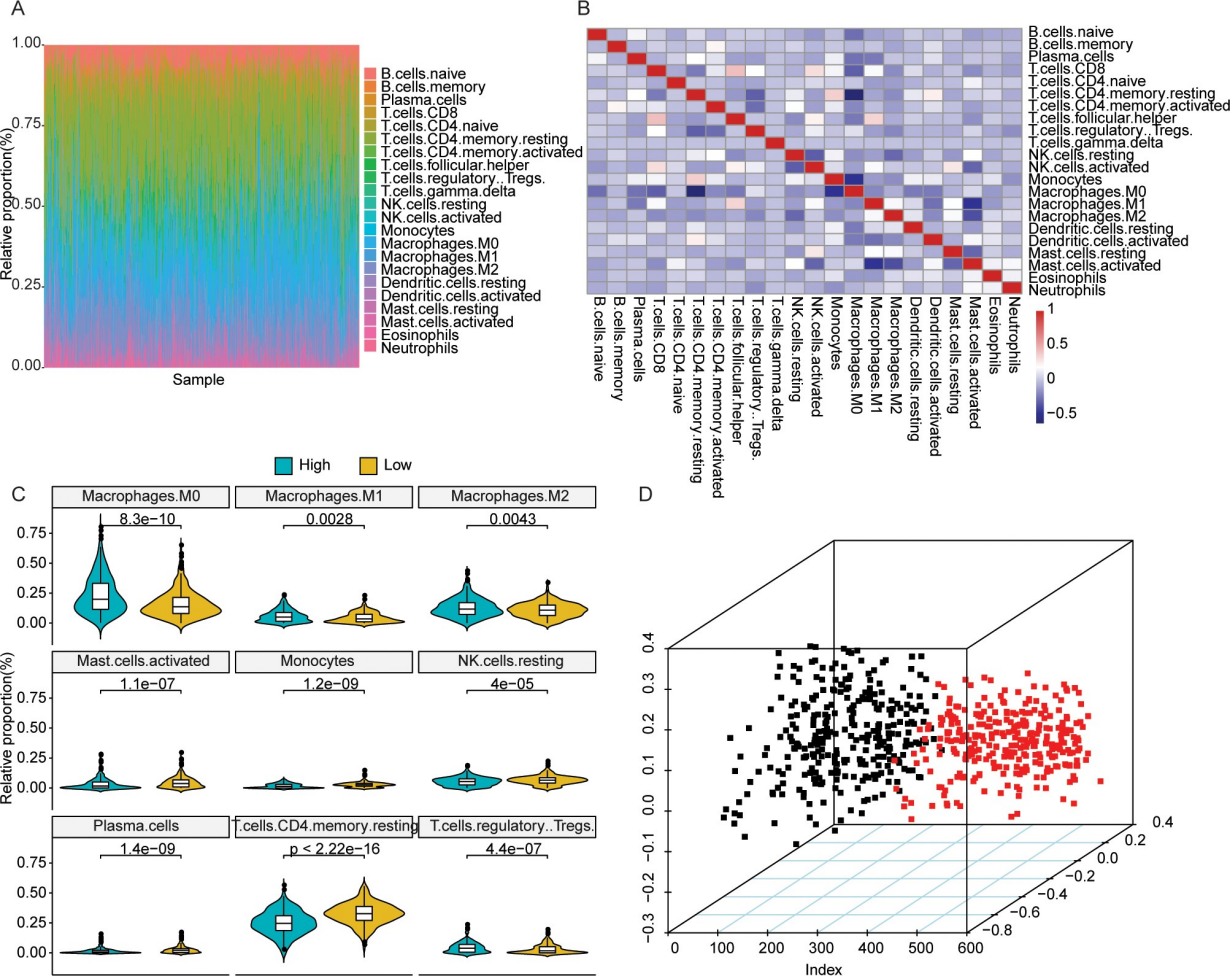
Immune infiltration of CRC patients with different risk scores. (A) Landscape of relative proportions of 22 TIICs across all of the CRC samples. (B) Correlation analysis of 22 TIICs. Red: positive correlation; Blue: negative correlation; Great correlation was represented by dark color. (C) Relative proportions of 9 TIICs exhibiting significantly different infiltrating degree between CRC samples with different risk scores. (D) PCA plot of CRC samples based on the relative proportions of the 9 significantly different TIICs.

As the changes in the proportion of TIICs in each CRC patient probably represented inherent characteristics, we estimated the 22 TIICs between low- and high-risk groups of CRC patients based on LM22 feature matrix. The immune cell infiltration analysis result of 590 CRC patients from TCGA dataset was shown in Fig 4A. No significant correlation was observed between different TIICs (Fig 4B), while 9 TIIC types exhibited significantly different relative proportions between CRC tumor samples with different risk scores (Fig 4C). Moreover, principle component analysis (PCA) based on those 9 TIICs could definitely separate the samples with high- and low-risk score (Fig 4D). Those results partially illustrated the potential association between the autophagy gene-based marker for CRC prognosis prediction and immune response.

**Fig 5:**
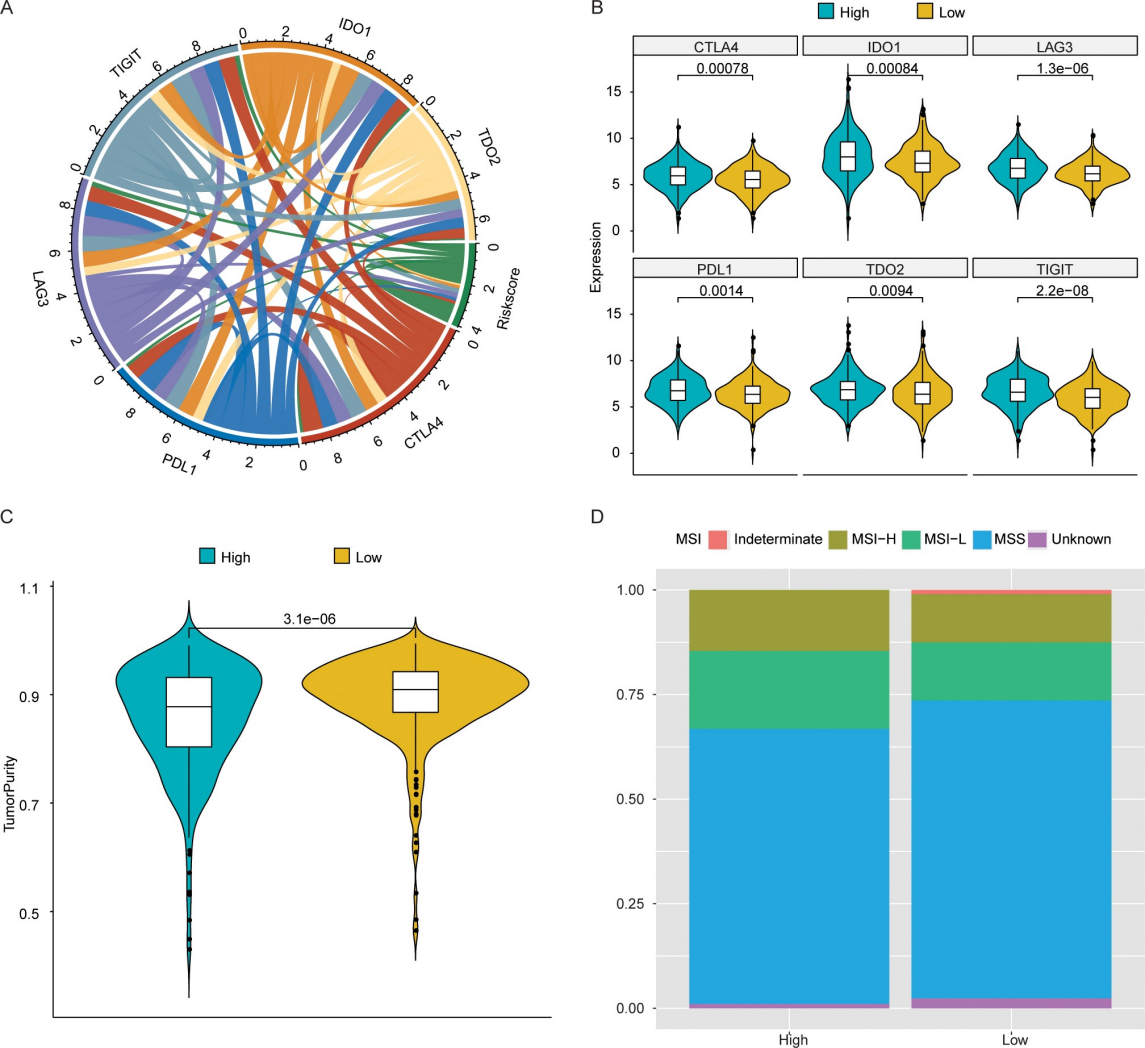
Association of risk score with several important immune checkpoints as well as tumor purity. (A) Chord diagram illustrating the correlations between mRNA levels of 6 key immune checkpoint receptors and the risk scores. The more lines between risk score and mRNAs, the greater the correlation. (B) Violin plots showing mRNA levels of the immune checkpoints which had obviously different expressions in low- and high-risk groups. (C) Violin plots showing the tumor purity in low- and high-risk groups. P value was obtained using Wilcoxon rank sum test. (D) The proportion of MSI-L + MSI-H in the high-risk group was higher than that in the low-risk group.

We further explored the associations between risk score and several vital immune checkpoint receptors expressions, which consisted of *CTLA4*, *PDL1*, *LAG3*, *TIGIT*, *IDO1* and *TDO2*. As a result, the mRNA levels of above immune checkpoint receptors were significantly associated with the risk score (Fig 5A), and the high-risk group presented obviously elevated expression of these immune checkpoint receptors compared with the low-risk group (Fig 5B). These results indicated that the inferior survival outcome in CRC patients with high risk score might be caused by the immunosuppressive microenvironment. Meanwhile, it was also found that compared with the low-risk group, the tumor purity in the high-risk group was obviously reduced (P value < 0.05) (Fig 5C). The MSI status in the two groups was also analyzed. As shown in Fig 5D, the proportion of MSI-L + MSI-H in the high-risk group was higher than that in the low-risk group.

## Discussion

Autophagy is a crucial self-digested process which removes the excessive or aberrant cellular components including organelles and proteins [14]. For the process of tumor occurrence and metastasis, autophagy has bidirectional regulation: On one hand, autophagy plays a key role in cell monitoring in case of transformation from normal cells to tumor cells. On the other hand, autophagy inhibits cellular death, offers enough nutrients, and induces drug resistance, which promotes tumorigenesis. [15]. The genes associated with autophagy are indicated to involve in accommodation of autophagy [7]. Numerous studies showed that, the expression of autophagy-related genes was potential biomarker for tumor surveillance. For instance, Gu YY. et al. reported that in breast cancer, an 8-gene based autophagy signature was able to forecast the distant metastasis-free survival [16]. Eissa S. et al. identified a urinary autophagy transcript signature consisted of 7 autophagy genes and validated the clinical application in bladder cancer patients [17].

Burada F. et al. named several autophagy-related genes in CRC, including *LC3*, *BECN1*, *ATG5*, *ATG10*, *AMBRA1*, *UVRAG*, *BCL2*, *Bif-1*, and inflammatory bowel disease susceptibility genes, however, the effects of these focused molecules in CRC is still unclear due to the conflicting results [7]. Herein, a total of 210 autophagy genes were included in this study, and 11 of them, i.e. *CX3CL1*, *ULK3*, *CDKN2A*, *NRG1*, *ATG4B*, *GAA*, *RGS19*, *DDIT3*, *GRID1*, *DAPK1* and *SERPINA1* were shown to be survival-related in CRC. Lacking the allele I249 in *CX3CR1* is able to partially affect CRC [18]. The tumor suppressor proteins p16 and p19ARF, corded by *CDKN2A*, was found to highly expressed in the CRC sections, however it seemed to be not directly related to colorectal carcinogenesis [19]. *ATG4B* is highly expressed in CRC cells. The mutation and promoter hypermethylation of *DAPK1* were related to generate diverse pathways of colorectal tumorigenesis [20]. The serpinA1 signaling was implicated in the processes including invasion and migration of CRC cells, and could be used as a promising biomarker for CRC prognosis and treatment [21]. To our knowledge, the other of these genes were proposed to be applied to the assessment of CRC prognosis for the first time. Thus, the specific association between these autophagy genes and CRC warrants further investigation. The multivariate Cox regression analysis showed the autophagy signature could independently predict CRC prognosis, which supported the accuracy of our above results to a certain extent. Wei et al. investigated the autophagy-related long noncoding RNAs (lncRNAs), screened 8 autophagy-related lncRNAs with prognostic value through univariate and multivariate Cox regression analysis, and constructed a predictive model based on these lncRNAs for CRC prognosis [22]. Based on 232 autophagy-related genes, Yang et al. identified 20 genes by using univariate Cox regression analysis and finally selected 8 genes *via* multivariate analysis, which were incorporated into the risk scoring model for the prognosis evaluation of CRC patients [23]. Zhou et al. identified the differentially expressed genes related to autophagy between the early relapse CRC patients and the long-term survival CRC patients, and selected 5 genes; With the multivariate Cox regression analysis, a 5-gene signature was established to assess the postoperative relapse of CRC patients [24]. Our study differs from these previous researches in the screening of candidate genes related to autophagy for the risk score model, which uses the LASSO Cox regression method. It may provide guidance for the optimization of candidate genetic markers in the establishment of prognostically predictive model for CRC.

Autophagy may promote the antitumor inflammatory responses, and suppress the metastasis of tumors in the early stage [25], while the immune microenvironment can also govern tumor progression. The tumor purity analysis showed that, the samples in high-risk group were more heterogeneous than that in low-risk group. Meanwhile, we analyzed contents of the immune cells infiltrated into tumor, and no significant correlation was found between the ratios of TIICs. However, several major immune cell subtypes, including Macrophages, mast cells, monocytes, NK cells, Plasma cells, T cells were significantly associated with the survival risk estimated by autophagy-related CRC signature. Those results offered the hypothesis that, the immue microenvironment in high-risk CRC patients were disturbing and thereby facilitated inferior clinical outcomes. Among the promising approaches that enhance anti-tumor immune responses, the blockade of immune checkpoint molecules is considered as the most effective one, which activates the anti-tumor immunity via “release the brakes” [26]. Our result also supported the associations of risk score with mRNA levels of several important immune checkpoint receptors, consisting of *CTLA4*, *PDL1*, *LAG3*, *TIGIT*, *IDO1* and *TDO2*, implying that this signature also had a certain hint on the effect of immunotherapy.

Collectively, a signature for CRC prognosis prediction was developed on the base of 11 autophagy genes, including *CX3CL1*, *ULK3*, *CDKN2A*, *NRG1*, *ATG4B*, *GAA*, *RGS19*, *DDIT3*, *GRID1*, *DAPK1* and *SERPINA1*. This autophagy-related signature is able to estimate the prognosis of CRC patients independently of other clinical factors and exhibits the potential as an immunotherapy signature in CRC. The signature can classify CRC patients and predict their progression as well as probability of certain outcomes. This may provide references for the prevention and treatment programs of CRC patients. To our knowledge, several genes in the risk score model are proposed to be used for the prognosis assessment of CRC patients for the first time. In addition, the LASSO Cox regression method may provide guidance for further optimization of candidate genetic markers in the predictive model for cancer prognosis. However, there are several limitations to our study. First, the datasets in our study are from publicly available databases and only in-silico approaches are used. Experimental test of the signature in the clinical CRC samples from our institute is lacking. This may affect the validation, reliability, and further application of the signature. We will devote more efforts to performing experimental investigation in the future. Secondly, the biological functions of the selected autophagy genes and the relationships between those genes with CRC are not fully investigated. Thirdly, only TCGA and GEO datasets are used in the analysis. More data from other databases would be helpful to support our results.

## Supporting information

S1 Fig The top 20 enriched GO terms (A) and KEGG pathways (B).
(TIF)

S1 Table A total of 210 autophagy genes. (DOCX)

S2 Table The results of functional enrichment analysis. (XLSX)
