# Supplementary figures and images for: A signature based on 11 autophagy genes for prognosis prediction of colorectal cancer
Source: PLoS One. 2021 Oct 26;16(10):e0258741. doi: 10.1371/journal.pone.0258741 (PMC8547631; doi:10.1371/journal.pone.0258741)

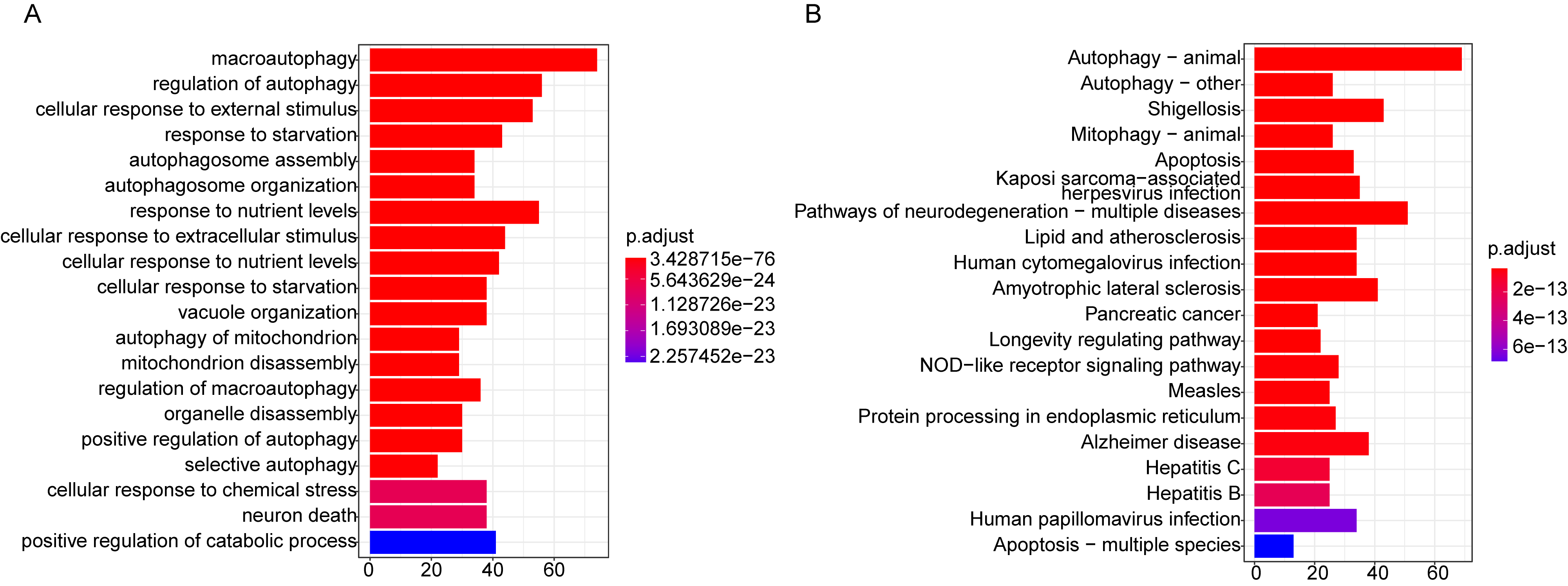

Supplement: S1 Fig — The top 20 enriched GO terms (A) and KEGG pathways (B). (TIF) [file pone.0258741.s001.tif]
