# Supplementary material for: A signature based on 11 autophagy genes for prognosis prediction of colorectal cancer
Source: PLoS One. 2021 Oct 26;16(10):e0258741. doi: 10.1371/journal.pone.0258741 (PMC8547631; doi:10.1371/journal.pone.0258741)
Supplement: S1 Table — (DOCX) [file pone.0258741.s002.docx]

Table S1

| Rank | GeneSymbol | Rank | GeneSymbol | Rank | GeneSymbol |
| --- | --- | --- | --- | --- | --- |
| 1 | CFLAR | 71 | NFKB1 | 141 | FOXO1 |
| 2 | ITGA3 | 72 | HSPA8 | 142 | ITPR1 |
| 3 | LAMP2 | 73 | ATG2A | 143 | BAG3 |
| 4 | CX3CL1 | 74 | AMBRA1 | 144 | HSPB8 |
| 5 | MBTPS2 | 75 | CAMKK2 | 145 | GRID2 |
| 6 | CAPN1 | 76 | CDKN1B | 146 | ATG10 |
| 7 | BID | 77 | IFNG | 147 | NRG1 |
| 8 | RB1CC1 | 78 | GAPDH | 148 | WIPI2 |
| 9 | FAS | 79 | PRKAB1 | 149 | NRG2 |
| 10 | BAK1 | 80 | VEGFA | 150 | PINK1 |
| 11 | PEX3 | 81 | ARSB | 151 | SQSTM1 |
| 12 | GABARAPL2 | 82 | BNIP1 | 152 | DIRAS3 |
| 13 | DAPK2 | 83 | KLHL24 | 153 | PEA15 |
| 14 | HSPA5 | 84 | EIF4G1 | 154 | IL24 |
| 15 | GOPC | 85 | BIRC6 | 155 | CAPN2 |
| 16 | VAMP3 | 86 | NFE2L2 | 156 | WDFY3 |
| 17 | MAPK9 | 87 | HDAC1 | 157 | MTMR14 |
| 18 | EIF2AK2 | 88 | CD46 | 158 | PRKCD |
| 19 | ATG5 | 89 | CTSD | 159 | CASP3 |
| 20 | NCKAP1 | 90 | STK11 | 160 | CTSB |
| 21 | CASP8 | 91 | ATF6 | 161 | DLC1 |
| 22 | GNAI3 | 92 | FOXO3 | 162 | TMEM74 |
| 23 | PRKCQ | 93 | FKBP1B | 163 | TSC1 |
| 24 | ATG2B | 94 | NKX2-3 | 164 | ZFYVE1 |
| 25 | SIRT2 | 95 | MAPK8IP1 | 165 | EEF2 |
| 26 | WIPI1 | 96 | CCR2 | 166 | MLST8 |
| 27 | TP63 | 97 | TNFSF10 | 167 | ATG16L2 |
| 28 | RAB7A | 98 | CXCR4 | 168 | FADD |
| 29 | MAP2K7 | 99 | VAMP7 | 169 | ATG4B |
| 30 | PIK3C3 | 100 | CDKN1A | 170 | RAB24 |
| 31 | TP53INP2 | 101 | ATG4C | 171 | SPNS1 |
| 32 | TP73 | 102 | CAPNS1 | 172 | GABARAP |
| 33 | SAR1A | 103 | BECN1 | 173 | FOS |
| 34 | ULK2 | 104 | CANX | 174 | KIF5B |
| 35 | CHMP2B | 105 | ATF4 | 175 | GAA |
| 36 | ATG16L1 | 106 | DNAJB9 | 176 | BCL2L1 |
| 37 | PPP1R15A | 107 | ATG4D | 177 | RGS19 |
| 38 | BAX | 108 | SESN2 | 178 | BCL2 |
| 39 | FKBP1A | 109 | DNAJB1 | 179 | PTEN |
| 40 | BIRC5 | 110 | RAF1 | 180 | RAB33B |
| 41 | NLRC4 | 111 | ITGB4 | 181 | EIF2AK3 |
| 42 | ITGA6 | 112 | EIF2S1 | 182 | RELA |
| 43 | HDAC6 | 113 | EDEM1 | 183 | DDIT3 |
| 44 | HSP90AB1 | 114 | DRAM1 | 184 | SPHK1 |
| 45 | SIRT1 | 115 | RAC1 | 185 | BNIP3 |
| 46 | SH3GLB1 | 116 | CALCOCO2 | 186 | ULK1 |
| 47 | MAPK1 | 117 | MYC | 187 | ERN1 |
| 48 | ARSA | 118 | CASP1 | 188 | ATG9B |
| 49 | APOL1 | 119 | RAB1A | 189 | GRID1 |
| 50 | ST13 | 120 | ATIC | 190 | TBK1 |
| 51 | HIF1A | 121 | GABARAPL1 | 191 | HGS |
| 52 | TM9SF1 | 122 | RB1 | 192 | P4HB |
| 53 | PTK6 | 123 | ULK3 | 193 | NRG3 |
| 54 | CHMP4B | 124 | MAP1LC3B | 194 | LAMP1 |
| 55 | MAP1LC3A | 125 | PELP1 | 195 | EIF4EBP1 |
| 56 | ATG4A | 126 | NPC1 | 196 | NBR1 |
| 57 | MAPK3 | 127 | TP53 | 197 | CLN3 |
| 58 | USP10 | 128 | RPTOR | 198 | PIK3R4 |
| 59 | TSC2 | 129 | ERBB2 | 199 | DAPK1 |
| 60 | EEF2K | 130 | CAPN10 | 200 | CASP4 |
| 61 | RAB11A | 131 | PEX14 | 201 | WDR45 |
| 62 | IKBKB | 132 | ARNT | 202 | SERPINA1 |
| 63 | BNIP3L | 133 | PARP1 | 203 | ATG7 |
| 64 | NAMPT | 134 | RAB5A | 204 | MAP1LC3C |
| 65 | RHEB | 135 | ATG3 | 205 | UVRAG |
| 66 | BAG1 | 136 | NAF1 | 206 | TUSC1 |
| 67 | MAPK8 | 137 | ATG12 | 207 | MTOR |
| 68 | RPS6KB1 | 138 | EGFR | 208 | ATG9A |
| 69 | CCL2 | 139 | CDKN2A | 209 | IRGM |
| 70 | PRKAR1A | 140 | ITGB1 | 210 | IKBKE |
